# Challenging trophic position assessments in complex ecosystems: Calculation method, choice of baseline, trophic enrichment factors, season and feeding guild do matter: A case study from Marquesas Islands coral reefs

**DOI:** 10.1002/ece3.11620

**Published:** 2024-06-30

**Authors:** Yves Letourneur, Pauline Fey, Jan Dierking, René Galzin, Valeriano Parravicini

**Affiliations:** ^1^ UMR ENTROPIE (UR‐IRD‐IFREMER‐CNRS‐UNC), Labex « Corail » Université de la Nouvelle‐Calédonie Nouméa Cedex New Caledonia; ^2^ GEOMAR Helmholtz Centre for Ocean Research Kiel Research Division Marine Ecology Kiel Germany; ^3^ CRIOBE, USR 3278 EPHE‐CNRS‐UPVD, LabEx « Corail », Université de Perpignan PSL Research University Perpignan Cedex France

**Keywords:** assessment methods, consumers, food webs, primary producers, trophic position

## Abstract

Assessments of ecosystem functioning are a fundamental ecological challenge and an essential foundation for ecosystem‐based management. Species trophic position (TP) is essential to characterize food web architecture. However, despite the intuitive nature of the concept, empirically estimating TP is a challenging task due to the complexity of trophic interaction networks. Various methods are proposed to assess TPs, including using different sources of organic matter at the base of the food web (the ‘baseline’). However, it is often not clear which methodological approach and which baseline choices are the most reliable. Using an ecosystem‐wide assessment of a tropical reef (Marquesas Islands, with available data for 70 coral reef invertebrate and fish species), we tested whether different commonly used TP estimation methods yield similar results and, if not, whether it is possible to identify the most reliable method. We found significant differences in TP estimates of up to 1.7 TPs for the same species, depending on the method and the baseline used. When using bulk stable isotope data, the choice of the baseline significantly impacted TP values. Indeed, while nitrogen stable isotope (δ^15^N) values of macroalgae led to consistent TP estimates, those using phytoplankton generated unrealistically low TP estimates. The use of a conventional enrichment factor (i.e. 3.4‰) or a ‘variable’ enrichment factor (i.e. according to feeding guilds) also produced clear discrepancies between TP estimates. TPs obtained with δ^15^N values of source amino acids (compound‐specific isotope analysis) were close to those assessed with macroalgae. An opposite seasonal pattern was found, with significantly lower TPs in winter than in summer for most species, with particularly pronounced differences for lower TP species. We use the observed differences to discuss possible drivers of the diverging TP estimates and the potential ecological implications.

## Abstract 2

Based on a case study from a remote coral reef ecosystem in the Pacific Ocean, this work analyses how several potential sources of organic matter (i.e. ‘baselines’) and calculation methods strongly affect assessments of trophic positions. We also demonstrated that seasonal temporality and feeding guild are important parameters to be considered.

## 1 INTRODUCTION

Studying food web structure and dynamics in an ecosystem is a complex challenge due to the multiplicity of functional groups and interactions between species (Hussey et al., 2014). The energetic flow through an ecosystem can be estimated by the use of discrete trophic levels, a concept derived from the theory of trophic dynamics (Lindeman, 1942). From this theory, a continuous quantitative measure of the hierarchical role of a given species in a food web has emerged, which is the trophic position (Hussey et al., 2014; Vander Zanden & Rasmussen, 1996). Thus, food webs can be viewed as consisting of functional groups (sensu trophic or feeding guilds) in which the trophic position (hereafter TP) of a species is measured on a continuous scale (Hussey et al., 2014). The concept of TP provides a standardized metric to better understand the structure and functioning of food webs, such as the length of food chains (Vander Zanden et al., 1999), the degree of omnivory (Thompson et al., 2007), the trophic cascades (Bascompte et al., 2005) and/or the alteration of trophic links (Vander Zanden & Rasmussen, 1999). This concept thus contributes to the description of trophic interactions within ecosystems allowing a better understanding of ecosystem functioning. By extension, it provides the foundation for the ecologically driven management of fisheries (Branch et al., 2010; Garcia et al., 2003; Pauly et al., 1998). Information about the TP of species and the trophic structure of an entire community also helps to assess the effects of anthropogenic and natural disturbances, as well as the persistence and resilience of food webs (Rooney et al., 2008).

However, while assigning TPs is relatively straightforward in theory, it can represent a substantial challenge in practice. Multiple methods and approaches have been proposed and applied, each with its own set of strengths and weaknesses (Nielsen et al., 2018). Historically, the method has mainly been the visual analysis of stomach contents to acquire information on the consumer's diet (Hyslop, 1980). However, this approach is time consuming and it is unrealistic to carry out such a work on all species of a highly diversified ecosystem. Additionally, stomach contents only represent the last meal ingested before sampling and thus only offer an immediate snapshot of the feeding process. To circumvent these limitations, many ecologists have turned to the use of TP data already acquired in other ecosystems and/or on species phylogenetically close to those of interest, for example through FishBase (Froese & Pauly, 2018), if the interest is directed towards fish. However, this solution also has weaknesses, because TPs provided by FishBase are of variable origins and reliability (Bierwagen et al., 2018), and for many species the estimates are based on semi‐quantitative diet data from limited time points that ignore potential seasonal fluctuations in feeding activity or differences among locations. An additional source of uncertainty in TP assessments can be due to intraspecific variability. For example, the trophic position of an individual (and *a fortiori* of a species) is a dynamic parameter, potentially changing with ontogeny, season and/or environment. Thus, assigning a unique, averaged TP value to a species is just a ‘mean’ theoretical representation to help in the food web understanding.

As an alternative to gut‐content analysis, nitrogen stable isotopes (δ^15^N) are commonly used to estimate the TPs of consumers. This approach (also known as ‘bulk’ stable isotope analysis, hereafter BSIA) is based on the principle that, in a consumer's tissues, the isotopes of nitrogen integrate the signature (sensu isotopic composition) of an organism's assimilated diet over time and space (Post, 2002a; Skinner et al., 2022; Vander Zanden & Rasmussen, 1999). These estimates are based on the assumption that the change in δ^15^N between prey and predator (i.e. tissue discrimination factor: Δ^15^N) is constant from the primary producer to top consumers, and that the TP of a consumer can thus be calculated by dividing the difference between its δ^15^N signature and the δ^15^N signature of the food web baseline by Δ^15^N. This entails that reliable estimates of Δ^15^N and knowledge of the baseline δ^15^N value are essential. Regarding Δ^15^N, the average factor of 3.4 ± 1.0‰ from one trophic level to the next is often used for aquatic organisms (Minagawa & Wada, 1984; Post, 2002a; Vander Zanden & Rasmussen, 2001). However, using this mean discrimination factor conceals the variations in Δ^15^N highlighted for certain taxa or trophic groups (Briand et al., 2016; Caut et al., 2009; Fey et al., 2021; Hussey et al., 2014; McCutchan et al., 2003), and neglects that discrimination is a dynamic process and not a constant one (Olive et al., 2003). Therefore, the use of a fixed Δ^15^N of 3.4‰ per trophic position is a frequent case, often for ‘practical reasons’ (it could be considered as the least bad proxy) or the lack of data for calculating the real Δ^15^N. Either way, it can generate significant biases in the quantification of the structure of the food web, for example by underestimating the TP of top predators and the length of the food chain (Hussey et al., 2014).

Additional bias in the BSIA method to estimate TPs can arise from difficulties in the estimation of the choice of the reference baseline (Post, 2002b). Choosing the major source(s) of organic matter (OM) as the baseline fuelling the food web is relatively common, but taking into account spatial and/or temporal variations in isotopic composition of primary producers remains a key and complex parameter (Briand et al., 2015; Fey et al., 2020, 2021). Additionally, in many complex ecosystems, consumers may rely on several, more or less contrasted baselines (Briand et al., 2016; Fey et al., 2021; Quezada‐Romegialli et al., 2018). Disentangling baselines in an important issue in all systems, including coral reefs, and that systematic differences between two (or more) potential sources of organic matter point to the fact that δ^13^C could be useful, combined with δ^15^N, in this regard in food web studies. To overcome some of these limitations, long‐lived primary consumers that are less prone to short‐term variability can be used as baselines (Cabana & Rasmussen, 1996; Post, 2002a; Vander Zanden & Rasmussen, 1999). The theoretical TP designated for primary consumers is 2 (vs. 1 for primary producers) but few studies have looked in detail at the diet of these taxa. In addition, some species classified as primary consumers may actually exhibit a certain degree of omnivory or feed on bacteria or detritus (Vander Zanden & Fetzer, 2007). Several studies have used zooplankton as a primary consumer (Hussey et al., 2014; McMahon et al., 2013); however, zooplankton can include omnivorous or even carnivorous organisms with contrasted size‐classes and their isotopic values are often higher than those of strict primary consumers (Lorrain et al., 2015). Fixed organisms such as filter‐feeders (oysters, mussels, etc.) or grazing gastropods appear to be a more reliable alternative and have been widely used in coastal and freshwater ecosystems (Cabana & Rasmussen, 1996; Layman et al., 2012; Post, 2002a).

The use of δ^15^N values of specific compounds (compound‐specific isotope analysis: CSIA), such as amino acids (AAs) (δ^15^N_AA_), isolated from consumer tissues, is another method for determining baselines and estimating TPs (Fey et al., 2021; Hannides et al., 2009; Houssard et al., 2017; Lorrain et al., 2015; Popp et al., 2007; Vander Zanden et al., 2013). Analysis of amino acids can greatly increase the interpretive power of bulk nitrogen isotope studies (Hannides et al., 2013). The advantage of using amino acids is that they will respond differently to trophic transfer (McClelland & Montoya, 2002). On one hand, some AAs called ‘source AAs’ remain relatively stable during the trophic transfer (for instance for phenylalanine, increase of approximately 0.4 ± 0.5‰ per trophic level of δ^15^N_Phe_; Chikaraishi et al., 2009). These AAs will therefore retain the isotopic value of the baseline, even when they are collected from consumers (Hannides et al., 2013). On the other hand, other AAs called ‘trophic AAs’ are markedly enriched in ^15^N at each trophic transfer, providing information on the TP of the consumer. For example, the δ^15^N of glutamic acid (δ^15^N_Glu_) can increase by 8.0 ± 1.2‰ between each trophic level (Chikaraishi et al., 2009).

Faced with the diversity of the different methods and/or parameters for evaluating TPs, there is a clear need for approaches that reduce the uncertainty around TP estimates. This is particularly important for comparing different species, different geographical areas or for modelling trophic processes in a given ecosystem. Here, using a case study of the complex coral reef ecosystem and food web of the Marquesas Islands, French Polynesia, we address this need by answering the following questions: (i) how strongly does the choice of method affect the resulting TP estimates? (ii) how strongly does the choice among possible alternative baselines and different trophic enrichment factors affect TP estimates? (iii) do the CSIAs give TP estimates that seem more realistic than the TPs obtained with BSIA methods? (iv) does seasonal variability affect TP assessments? To answer the first question, we compared TPs calculated from different published equations. To answer the second question, the δ^15^N values of different baselines were used in combination with different enrichment factors' values, either primary producers or primary consumers. To answer the third question, the δ^15^N_AA_ values of several mesopredators were measured and then used for calculation. Finally, to answer the fourth question, we compared TPs of a subset of species sampled in both winter and summer.

## 2 MATERIALS AND METHODS

### 2.1 Site, sampling and studied species

**FIGURE 1:**
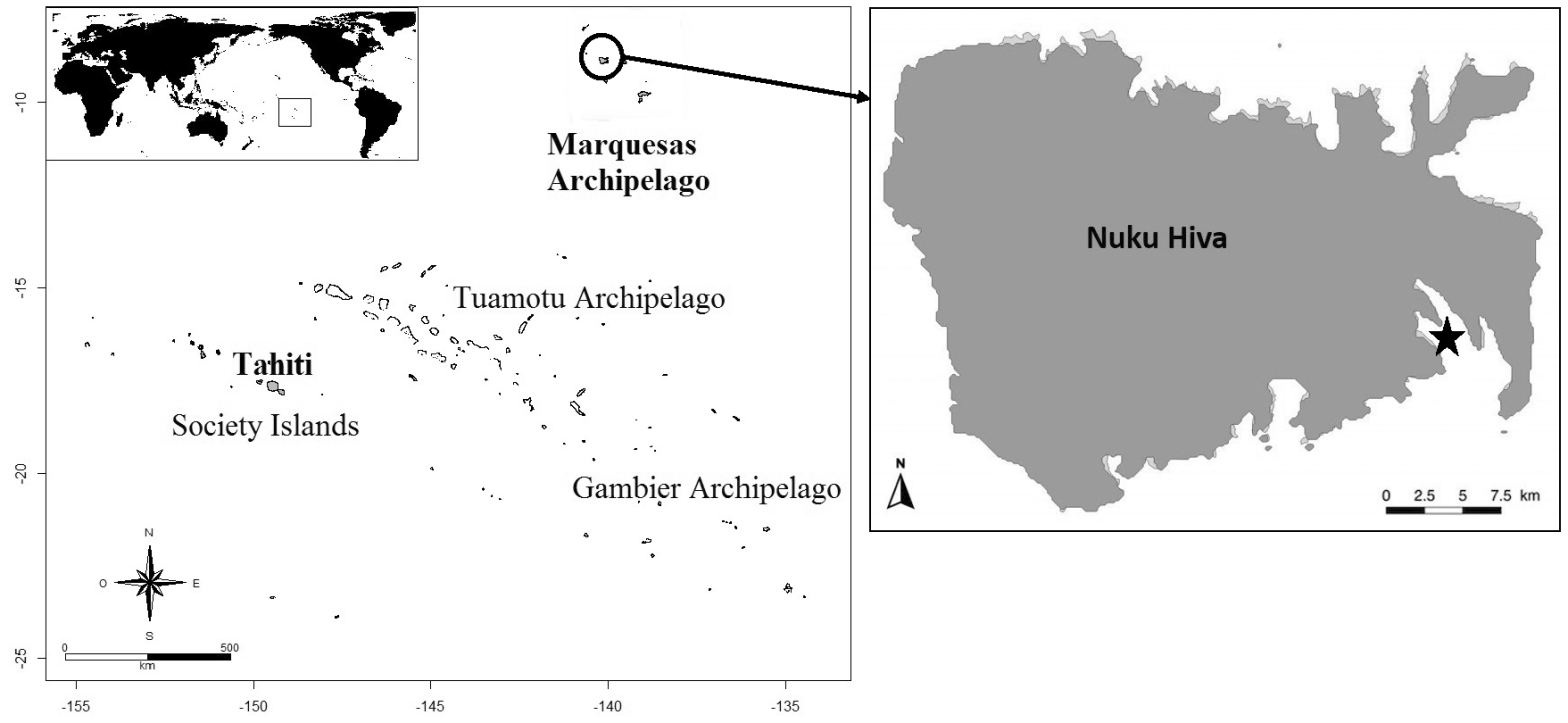
Location of the sampled area, that is, the ‘“Baie du Contrôleur’ (black star), in Nuku Hiva, Marquesas Islands, French Polynesia.

The data set for the case study for the method comparison was obtained in Nuku Hiva (Figure 1), the largest of the Marquesas Islands (8°54′ S, 140°02′ W), French Polynesia. Major local environmental characteristics and the sampling methods were already described in detail in previous studies (Fey et al., 2020, 2021; Galzin et al., 2016). Briefly, the studied area, named the ‘Baie du Contrôleur’, has relatively strong hydrodynamic conditions, and hosts a marine seafloor dominated by rocky habitats, characterized mainly by steep scree slopes of volcanic rock mixed with patches of soft‐bottom habitats, algae groves, coral habitats and caves. The benthic community is composed mainly of algal turf, macroalgae, scattered coral colonies and sponges. Other distinctive features of the studied site include the absence of *Acropora* spp. corals, which are common across other Polynesian coral reefs, and a mean live coral cover of only ~5%. Sampling was realized at two seasons, in August 2016 (austral winter) and March 2017 (summer).

Among the various potential sources of organic matter fuelling the food web (Fey et al., 2020), phytoplankton and macroalgae were overall the most important sources of organic matter in this system (Fey et al., 2021) and were thus considered for the analyses in the present study (phytoplankton: *n* = 39; macroalgae: *n* = 71). Among primary consumers, molluscs (gastropods and bivalves) are usually assumed to integrate the baseline with little spatiotemporal fluctuation (Cabana & Rasmussen, 1996; Layman et al., 2012; Post, 2002a). We therefore used the grazing gastropod *Mauritia* spp. (*n* = 18) and the filter‐feeder oyster *Pinctada margaritifera* (*n* = 24) as primary consumers for baseline calculations. Other primary consumers and several secondary–tertiary consumers (invertebrates and fish; *n* = 3–43, depending on the species for a total of 737 individuals analysed) were sampled to assess their TPs. Among secondary–tertiary consumers, we also selected eight mesopredator species expected to be at the top of the local benthic food webs for a compound‐specific stable isotope analysis (CS‐SIA) (see below). These species were one gastropod, *Conus conco*, and seven fish: the snappers *Lutjanus bohar*, *Lutjanus gibbus* and *Lutjanus kasmira*, the moray‐eel *Enchelycore pardalis*, the scorpionfish *Scorpaenopis possi* and the groupers *Cephalopholis argus* and *Epinephelus fasciatus* (*n* = 6 for each species, except *L. bohar*, *n* = 4).

Invertebrates were collected by handpicking during scuba diving, and fish were collected by spearfishing or using an anaesthetic (i.e. eugenol diluted at 10% in alcohol), both in winter and summer. For most animal organisms (total of 70 species), tissues analysed were muscles and, for each taxonomic group, systematically the same location (e.g. dorsal muscle in fish, abductor muscle in bivalves, etc.). For ascidians and sponges, ~5–10 g pieces, excluding external theca for ascidians, were taken from each individual.

### 2.2 Stable isotope analyses

#### 2.2.1 Bulk SIA

Animal tissues (muscles or small pieces of organisms, see above) were taken and immediately frozen at −20°C for subsequent analyses. Tissue samples of macro‐invertebrates and fish were freeze‐dried and ground to fine powder with a porcelain mortar and pestle. Approximately 1 mg of powder was weighed and encapsulated in tin caps. The bulk δ^15^N values were determined using continuous‐flow isotope‐ratio mass spectrometry with a Flash 2000 elemental analyser equipped with the Smart EA option (Thermo Scientific, Milan, Italy), coupled with a Delta V Advantage isotope‐ratio mass spectrometer with a ConFlo IV interface (Thermo Scientific, Bremen, Germany) at the Littoral, Environment and Societies Joint Research Unit stable isotope facility (LIENSs) at the University of La Rochelle (France). Calibration was done using reference materials (USGS‐61, ‐62, IAEAN2, –NO–3, – 600 for nitrogen). The analytical precision of the measurements was <0.15‰ based on analyses of USGS‐61 and USGS‐62 used as laboratory internal standards.

#### 2.2.2 Compound‐specific SIA

For δ^15^N_AA_ analyses, samples were prepared by acid hydrolysis followed by derivatization to produce trifluoroacetic amino acid esters (TFAAs) using a standard method (Popp et al., 2007). The δ^15^N values of the TFAA derivatives of amino acids were analysed using an isotope‐ratio mass spectrometer (Delta V Plus, Thermo Scientific, Bremen, Germany) interfaced with a gas chromatograph (GC) (Trace GC 1300, Thermo Scientific, Bremen, Germany) through a GC IsoLink combustion furnace, and liquid nitrogen cold trap at the University of Davis (California, USA). Measured isotopic values were corrected relative to known δ^15^N values of norleucine, the internal reference material. All samples were analysed in triplicate. Average standard deviation (SD) of triplicate measurements was no greater than ±1.25 across amino acids (within sample/reference materials) and across samples (within amino acids). Standard deviation of individual amino acids within sample/reference materials was no greater than ±1.75.

### 2.3 Methods for TP assessments

#### 2.3.1 FishBase references

For fish, we considered trophic positions defined from diet studies and listed in the database ‘FishBase’ (Froese & Pauly, 2018). We cannot do the same for invertebrates because, to the best of our knowledge, no equivalent of FishBase gives trophic positions for such species, although their putative feeding categories can be estimated. The fish TPs can be used as reference values and are named TP_Ref_ hereafter. Despite the variable origins and reliability (Bierwagen et al., 2018) and sometimes more or less arbitrary TPs, these data may provide useful information on the diet of fish and are used in several studies (e.g. Nielsen et al., 2015; Page et al., 2013). Fish length is a potentially important parameter for TP, and we then sampled adult individuals whose size is consistent with the common fish size found in FishBase.

#### 2.3.2 δ ^15^N bulk SIA method

Another simple and widely used model based on the use of BSIA values for estimating the TPs of various consumers was proposed by Post (2002a):

$${\mathrm{TP}}_{\text{consu}}=\left(\frac{\left(\delta^{15}N_{\text{consu}}-\delta^{15}N_{\text{base}}\right)}{\mathrm{\Delta N}}\right)+{\mathrm{TP}}_{\text{base}}$$

where TP_consu_ is the TP of the studied consumer and δ^15^N_consu_ its average nitrogen isotopic composition. The δ^15^N_base_ comes from a reference organism (the baseline) whose TP_base_ is the TP defined according to the trophic compartment to which it belongs (TP_base_ = 1 for primary producers, TP_base_ = 2 for primary consumers). ΔN corresponds to the enrichment factor of δ^15^N. Two ways were explored for ΔN, i.e. using the conventional value of 3.4‰ (Post, 2002a), and considering ‘variable’ trophic enrichment factors depending on trophic categories. Based on results from Fey et al. (2021), we defined trophic enrichment factors of 2.2‰ for filter‐feeders, 3.0‰ for zooplanktivores, 4.3‰ for herbivores–detritivores, and 2.5‰ for carnivores, and the value of 3.4‰ was maintained for omnivores. Those different trophic enrichment factors' values well reflect the differences found between phytoplankton and filter‐feeders, between phytoplankton and zooplankton, between macroalgae/turf and herbivores, and between herbivores/omnivores and carnivores, respectively (Fey et al., 2021).

Two primary producers were used here as baselines, i.e. macroalgae and phytoplankton (Table 1, Fey et al., 2020). The TPs of the different invertebrate and fish species obtained with these primary producers are noted as TP_algae_ and TP_phyto_ in the Results section using the conventional 3.4‰ value, and TP_algae‐va_ and TP_phyto‐va_ in the Results section using the variable trophic enrichment factors. The two primary consumers used are *Mauritia* spp. (i.e. *Mauritia mauritiana* and *Mauritia maculifera*, which were pooled due to low sample size and an absence of significant differences in their respective isotopic signatures) and *P. margaritifera* (Table 1, Fey et al., 2021). TPs obtained with these primary consumers are noted as TP_Masp_ and TP_Pima_ in the Results section using the conventional 3.4‰ value, and TP_Masp‐va_ and TP_Pima‐va_ in the Results section using the variable trophic enrichment factors.

**TABLE 1:** Baseline δ^15^N values, expressed in ‰, used for TP calculation (A) for bulk SIA or with δ^15^N_AA‐Sr_, and (B) for seasonal variations.

	(A)	(B)	
	Bulk	Winter	Summer
Primary producers			
Macroalgae	11.6	10.2	11.9
Phytoplankton	15.0	13.1	16.5
Primary consumers			
*Mauritia* spp.	16.3	15.8	16.6
*Pinctata margaritifera*	14.7	15.2	14.5
δ^15^N_AA‐Sr_	11.6	12.8	10.5

#### 2.3.3 ‘Classical’ analytical method with δ ^15^N_AA_ _sources_

The isotopic analysis of the amino acids of eight mesopredators made it possible to obtain an estimate of the δ^15^N value of the baseline, thanks to the use of source AAs. The δ^15^N_AA‐Sr_ values were calculated by averaging the δ^15^N values of phenylalanine and glycine, which are the recommended source AAs in TP estimates (Chikaraishi et al., 2009; Ohkouchi et al., 2017). These δ^15^N_AA‐Sr_ values were then applied as a baseline to the formula of Post (2002a) mentioned above (Table 1, Fey et al., 2021). The TPs estimated with this approach are noted TP_AA‐Sr_ in the Results section using the conventional 3.4‰ value and TP_AA‐Sr‐va_ using the variable trophic enrichment factors.

#### 2.3.4 Combining source and trophic amino acid δ ^15^N_AA_ values

Several studies have used the δ^15^N_AA_ values of consumers to calculate TPs, all of them applying the equation proposed by Chikaraishi et al. (2009) for this purpose:

$${\mathrm{TP}}_{\mathrm{Tr}-\mathrm{Sr}}=\frac{\left(\delta^{15}N_{\mathrm{AA}-\mathrm{Tr}}-\delta^{15}N_{\mathrm{AA}-\mathrm{Sr}}-\beta_{\mathrm{Tr}-\mathrm{Sr}}\right)}{\Delta_{\mathrm{Tr}-\mathrm{Sr}}}+1$$

where *β*_Tr‐Sr_ is the difference between the δ^15^N values of trophic AA (AA‐Tr) and sources AA (AA‐Sr) in primary producers and ∆_Tr‐Sr_ is the enrichment factor between AA‐Tr and AA‐Sr. Chikaraishi et al. (2009) suggest to employ the values of glutamic acid (trophic) and phenylalanine (source) for this calculation, due to their relatively large and constant ^15^N enrichment in Glu compared to Phe (Δ_Glu‐Phe_ = 7.6‰ and *β*_Glu‐Phe_ = 3.4‰). The TPs calculated for the eight mesopredators with these constants are designated as TP_Glu‐Phe_(1) hereafter. Other studies, based on a larger number of samples than Chikaraishi et al. (2009), recommend using the constants Δ_Glu‐Phe_ = 6.6‰ and *β*_Glu‐Phe_ = 2.8‰ (Nielsen et al., 2015; Sackett et al., 2015). The TPs estimated with these constants, also for the eight studied mesopredators, are designated as TP_Glu‐Phe_(2) hereafter.

However, several studies suggest that calculations of trophic positions based on multiple values of δ^15^N of several trophic and source AAs (i.e. not only glutamic acid and phenylalanine) would improve the estimation (Bradley et al., 2014; Choy et al., 2015; Décima et al., 2013; Hannides et al., 2013; Houssard et al., 2017). Thus, the combinations of δ^15^N values of several source AAs (glycine [Gly], phenylalanine [Phe]) and trophic AAs (alanine [Ala], glutamic acid [Glu], leucine [Leu], proline [Pro]) are also used (δ^15^N_AA‐Tr_ = 31.2‰; Fey, 2019). Regarding these amino acid combinations, the constants used for the mesopredators' TP estimates are: Δ_Tr‐Sr_ = 5.7‰ and *β*_Tr‐Sr_ = 3.6‰ (Choy et al., 2015; Houssard et al., 2017). These estimates of TPs of the eight studied mesopredators are designated as TP_Tr‐Sr_ hereafter.

### 2.4 Assessment of the effects of seasonal fluctuations

Marquesas Islands show a strong seasonal variation that produces remarkable differences in the influence of major energetic pathways, i.e. phytoplankton and macroalgae, among seasons (Fey et al., 2020; Galzin et al., 2016), which is reflected at the level of species isotopic composition (Fey et al., 2021). To test whether seasonal fluctuation may affect the estimation of trophic positions, we performed our analyses using the δ^15^N values corresponding to each season, i.e. winter versus summer, for primary producers, primary consumers and for δ^15^N_AA‐Sr_ values (Table 1). Statistical significance of seasonal differences was assessed with non‐parametric Kruskal–Wallis test.

## 3 RESULTS

### 3.1 Differences between TP assessment methods

The application of different methods to obtain TPs yielded substantially different estimates, depending on the specific calculation formulae and baseline that were chosen (Table 2). Overall, for all consumers analysed, the lowest TP values were found with phytoplankton as the baseline (TP_phyto_ or TP_phyto‐va_ depending on species). The highest TP values were found with *P. margaritifera* as primary consumer for the baseline (TP_Pima_), macroalgae as baseline with variable enrichment factors (TP_algae‐va_) or sometimes with source AAs (TP_AA‐Sr‐va_) depending on species (Table 2). Strong differences in TPs assessed with different methods were found, up to ~1.7 TP for sponges and Muricidae for instance. Overall, for all consumers, the range between TPs obtained with the two primary producers (TP_phyto_ and TP_algae_) was ~1.0 but decreased to ~0.8 when considering variable trophic enrichment factors (TP_phyto‐va_ and TP_algae‐va_), and the range between the two primary consumers (TP_Masp_ and TP_Pima_) was ~0.5 but it also decreased to ~0.3–0.4 when considering variable trophic enrichment factors (TP_Masp‐va_ and TP_Pima‐va_) (Table 2). Results obtained with algae and source AAs as potential baselines (TP_algae_ and TP_AA‐Sr_, or TP_algae‐va_ and TP_AA‐Sr‐va_) were the closest in all cases. Overall, considering variable trophic enrichment factors rather than the conventional 3.4‰ value generated a decrease of TPs of ~0.3–0.4 for herbivores (*Acanthurus* spp., *Scarus* spp., etc.) and an increase of TPs up to ~0.8–0.9 for carnivores (Scorpaenidae, Carangidae, etc.) (Table 2).

**TABLE 2:** Mean trophic positions (± SD) calculated with different methods and different trophic enrichment factors (see text).

(A)		TP_algae_	TP_algae‐va_	TP_phyto_	TP_phyto‐va_	TP_Masp_	TP_Masp‐va_	TP_Pima_	TP_Pima_va_	TP_AA‐Sr_	TP_AA‐Sr‐va_	TP_ref_
Acanthuridae	*Acanthurus lineatus*	2.47 ± 0.18	2.16	1.45	**1.36**	2.08	2.06	**2.56**	2.44	2.45	2.15	2.0 ± 0.0
	*Acanthurus nigricans*	2.45 ± 0.29	2.14	1.43	**1.34**	2.06	2.04	**2.53**	2.42	2.43	2.13	2.0 ± 0.0
	*Acanthurus pyroferus*	2.95 ± 0.27	2.54	1.93	**1.73**	2.55	2.44	**3.03**	2.82	2.93	2.53	2.0 ± 0.0
	*Acanthurus reversus*	2.33 ± 0.16	2.05	1.31	**1.25**	1.94	1.95	**2.41**	2.33	2.31	2.04	2.1 ± 0.1
	*Acanthurus triostegus*	2.59 ± 0.11	2.26	1.59	**1.46**	2.20	2.16	**2.68**	2.54	2.58	2.25	2.8 ± 0.4
	*Ctenochaetus flavicauda*	3.17 ± 0.31	2.71	2.15	**1.91**	2.77	2.61	**3.25**	2.99	3.15	2.70	nd
	*Ctenochaetus marginatus*	3.03 ± 0.42	2.61	2.02	**1.80**	2.64	2.51	**3.11**	2.89	3.02	2.59	2.0 ± 0.0
Apogonidae	*Apogon lativittatus*	3.63 ± 0.32	3.98	**2.61**	2.82	3.23	3.40	3.71	3.94	3.61	**3.96**	3.4 ± 0.4
	*Ostorhinchus relativus*	3.35 ± 0.15	3.66	**2.33**	2.51	2.96	3.08	3.43	3.62	3.33	**3.64**	3.5 ± 0.5
Blenniidae	*Cirripectes variolosus*	2.62 ± 0.32	2.28	1.60	**1.47**	2.22	2.18	**2.70**	2.55	2.60	2.26	2.0 ± 0.0
Caesionidae	*Pterocaesio marri*	3.10 ± 0.08	**3.38**	**2.08**	2.23	2.71	2.80	3.18	3.34	3.08	3.36	nd
Carangidae	*Trachinotus* sp.	3.31 ± 0.08	**4.14**	**2.29**	2.75	2.91	3.24	3.39	3.89	3.29	4.11	3.6 ± 0.5
Chaetodontidae	*Chaetodon citrinellus*	**3.44** ± 0.29		**2.43**		3.05		3.53		3.43		3.5 ± 0.3
	*Chaetodon trichrous*	**3.45** ± 0.15		**2.43**		3.06		3.53		3.43		3.3 ± 0.6
Cirrhitidae	*Cirrhitichthys oxycephalus*	3.22 ± 0.31	**4.03**	**2.21**	2.64	2.83	3.13	3.31	3.78	3.21	4.00	4.0 ± 0.7
Holocentridae	*Myripristis berndti*	2.97 ± 0.24	**3.23**	**1.95**	2.08	2.58	2.66	3.06	3.20	2.95	3.21	3.7 ± 0.6
	*Myripristis earlei*	2.79 ± 0.38	**3.03**	**1.77**	1.87	2.39	2.45	2.87	2.99	2.77	3.01	3.5 ± 0.5
	*Myripristis pralinia*	2.89 ± 0.06	**3.14**	**1.87**	1.98	2.49	2.56	2.97	3.10	2.87	3.12	3.5 ± 0.5
	*Sargocentron punctatissimum*	3.24 ± 0.18	**3.54**	**2.23**	2.39	2.85	2.97	3.32	3.51	3.22	3.52	3.4 ± 0.4
	*Sargocentron tiere*	3.37 ± 0.10	**3.69**	**2.36**	2.54	2.98	3.11	3.46	3.65	3.36	3.67	4.2 ± 0.7
Labridae	*Halichoeres claudia*	3.43 ± 0.10	4.03	**2.41**	2.92	3.04	3.41	3.51	4.06	3.41	**4.28**	3.4 ± 0.5
	*Thalassoma amblycephalum*	2.91 ± 0.08	3.60	**1.90**	2.22	2.52	2.71	3.00	3.36	2.89	**3.58**	3.1 ± 0.2
Lethrinidae	*Monotaxis grandoculis*	3.88 ± 0.01	4.29	**2.86**	3.53	3.49	4.02	3.96	4.67	3.86	**4.89**	3.4 ± 0.0
Microdesmidae	*Ptereleotris zebra*	2.79 ± 0.29	3.44	**1.77**	2.05	2.40	2.55	2.88	3.19	2.77	**3.41**	3.4 ± 0.5
Muraenidae	*Gymnothorax buroensis*	3.32 ± 0.26	**4.15**	**2.30**	2.77	2.93	3.26	3.41	3.91	3.30	4.13	3.8 ± 0.7
	*Uropterygius xanthopterus*	2.79 ± 0.20	**3.44**	**1.78**	2.05	2.40	2.55	2.88	3.19	2.78	3.41	3.5 ± 0.6
Pomacanthidae	*Centropyge flavissima*	3.12 ± 0.21		**2.11**		2.73		**3.21**		3.11		2.8 ± 0.3
Pomacentridae	*Chromis abrupta*	3.20 ± 0.27	**3.50**	**2.18**	2.34	2.81	2.92	3.29	3.46	3.18	3.48	3.0 ± 0.1
	*Chromis flavapicis*	3.21 ± 0.07	**3.51**	**2.19**	2.35	2.82	2.93	3.30	3.47	3.19	3.49	3.4 ± 0.4
	*Dascyllus strasburgi*	3.46 ± 0.29	**3.79**	**2.45**	2.64	3.07	3.22	3.55	3.76	3.45	3.77	3.0 ± 0.4
	*Lepidozygus tapeinosoma*	2.85 ± 0.26	**3.10**	**1.83**	1.95	2.46	2.52	2.94	3.06	2.83	3.08	3.4 ± 0.5
	*Pomacentrus coelestis*	2.49 ± 0.14	2.18	1.47	**1.37**	2.10	2.08	**2.58**	2.46	2.47	2.17	3.2 ± 0.3
Scaridae	*Scarus koputea*	2.66 ± 0.46	2.31	1.64	**1.50**	2.26	2.21	**2.74**	2.59	2.64	2.30	nd
	*Scarus rubroviolaceus*	3.13 ± 0.33	2.68	2.11	**1.88**	2.73	2.58	**3.21**	2.96	3.11	2.67	2.0 ± 0.0
Scorpaenidae	*Pterois antennata*	3.52 ± 0.14	**4.43**	**2.50**	3.04	3.13	3.54	3.61	4.18	3.50	4.40	3.6 ± 0.6
	*Scorpaenodes evides*	2.65 ± 0.25	**3.24**	**1.63**	1.86	2.26	2.35	2.73	3.00	2.63	3.22	4.2 ± 0.7
Serranidae	*Epinephelus irroratus*	2.80 ± 0.16	**3.45**	**1.78**	2.73	2.41	2.56	2.89	3.20	2.78	3.42	3.7 ± 0.6
Tripterygiidae	*Enneapterygius rhabdotus*	2.65 ± 0.11	**3.24**	**1.63**	1.85	2.25	2.35	2.73	2.99	2.63	3.21	3.1 ± 0.3

(B)		TP_algae_	TP_algae‐va_	TP_phyto_	TP_phyto‐va_	TP_Masp_	TP_Masp‐va_	TP_Pima_	TP_Pima_va_	TP_AA‐Sr_	TP_AA‐Sr‐va_	Feeding category
Ascidiidae	*Ascidia* sp.	2.00 ± 0.31	**2.55**	0.99	**0.98**	1.61	1.40	2.09	2.14	1.99	2.52	FF
Buccinidae	*Latirus nodatus*	2.86 ± 0.08	**3.53**	**1.84**	2.15	2.47	2.64	2.95	3.29	2.84	3.51	C
Diadematidae	*Echinothrix diadema*	2.39 ± 0.45	2.10	1.37	**1.29**	2.00	2.00	**2.48**	2.38	2.37	2.09	HO
Diogenidae	*Ciliopagurus vakovako*	2.08 ± 0.23		**1.06**		1.69		**2.16**		2.06		OD
	*Dardanus sanguinocarpus*	1.98 ± 0.23		**0.96**		1.59		**2.06**		1.96		OD
Holothuridae	*Holothuria* sp.	2.96 ± 0.22		**1.95**		2.57		**3.05**		2.94		D
Muricidae	*Chicoreus maurus*	3.04 ± 0.12	**3.78**	**2.03**	2.40	2.65	2.89	3.13	3.54	3.03	3.76	C
	*Chicoreus ramosus*	3.01 ± 0.19	**3.74**	**2.00**	2.36	2.62	2.85	3.10	3.50	3.00	3.72	C
	*Drupa ricinus*	3.02 ± 0.14	**3.75**	**2.00**	2.36	2.63	2.86	3.11	3.50	3.00	3.72	C
	*Mancinella armigera*	3.36 ± 0.28	4.12	**2.35**	2.83	2.97	3.32	3.45	3.97	3.35	**4.19**	C
Octopodidae	*Octopus* cf. *cyanea*	2.51 ± 0.24	**3.05**	**1.49**	1.67	2.12	2.16	2.59	2.81	2.49	3.03	C
Ophiocomidae	*Macrophiothrix propinqua*	2.65 ± 0.02		**1.64**		2.26		**2.74**		2.64		D
	*Ophiarthrum elegans*	2.76 ± 0.29		**1.74**		2.37		**2.84**		2.74		D
	*Ophiocoma* cf. *cynthiae*	2.32 ± 0.32		**1.30**		1.92		**2.40**		2.30		D
	*Ophiocoma erinaceus*	2.71 ± 0.10		**1.70**		2.32		**2.80**		2.70		D
Oreasteridae	*Culcita novaeguineae*	2.26 ± 0.24		**1.24**		1.87		**2.35**		2.24		O
Palaemonidae	*Periclimenes soror*	2.73 ± 0.06		**1.71**		2.34		**2.81**		2.71		O
Pinnidae	*Streptopinna saccata*	1.94 ± 0.06	**2.45**	0.92	**0.88**	1.54	1.30	2.02	2.04	1.92	2.30	FF
Pteriidae	*Pteria macroptera*	2.48 ± 0.10	**3.29**	**1.47**	1.72	2.09	2.14	2.57	2.88	2.47	3.26	FF
Sponges	*Sponge* sp.1	2.40 ± 0.11	**3.16**	**1.38**	1.59	2.01	2.02	2.48	2.75	2.38	3.13	FF
	*Sponge* sp.2	2.41 ± 0.05	**3.18**	**1.39**	1.60	2.02	2.03	2.49	2.76	2.39	3.15	FF
	*Sponge* sp.3	2.08 ± 0.29	**2.66**	**1.06**	1.09	1.68	1.51	2.16	2.25	2.06	2.64	FF
	*Spheciospongia* sp.	2.19 ± 0.30	**2.85**	**1.18**	1.28	1.81	1.70	2.28	2.44	2.18	2.82	FF
Toxopneustidae	*Tripneustes gratilla*	2.22 ± 0.28	1.97	1.21	**1.16**	1.83	1.87	**2.31**	2.24	2.21	1.95	HO

*Note*: For fish species (A), the mean trophic positions given by FishBase (TP_ref_) are also given (nd = no data); the cases of the eight mesopredators are shown in Figure 2. For invertebrates (B), putative feeding categories are given as follows: C, carnivore; D, detritivore; FF, filter‐feeder; HO, herbivore‐omnivore; O, omnivores. Data from both seasons are pooled. As standard deviation (SD) values are the same for each species irrespective of the calculation method, they are given only in the first column, i.e. TP_algae_. SD values of 0.0 in TP_ref_ (FishBase) indicate that all TPs given in that database give the same TP. For each species, the values in bold characters indicate the minimum and maximum estimated TPs (excluding FishBase).

We found several unrealistic results for TPs, sensu those values were lower than 2, that is the minimal theoretical value for the TP of an exclusively herbivorous (or filter‐feeder) species feeding only on phytoplankton or algae. These results (i.e. TPs <2) mostly concerned TP_phyto_ and/or TP_phyto‐va_ values for both fish and invertebrates (Table 2). TPs <2 were also found for some invertebrates with *Mauritia* spp. and for a few species with source AAs as potential baselines (Table 2).

Specifically for fish, the data extracted from FishBase (TP_ref_) were in disagreement with some of our results. For instance, we found a remarkable difference between TP_AA‐Sr_ and TP_ref_ given for several species, including *Acanthurus* spp., *Ctenochaetus marginatus*, *Sargocentron tiere*, Muraenidae, *Scarus rubroviolaceus* or *Scorpaenodes evides*, although differences were less pronounced with variable trophic enrichment factors (TP_Ref_ vs. TP_AA‐Sr‐va_) (Table 2). Conversely, some TP_ref_ values were close to TP_AA‐Sr_ values, such as for Chaetodontidae, *Halichoeres claudia* or *Pterois antennata*, but values were less close when considering TP_AA‐Sr‐va_ values for the two latter species.

Invertebrate comparisons were difficult due to the lack of TP_ref_ values for such species. However, comparing TP_AA‐Sr_ (or TP_AA‐Sr‐va_) and putative feeding categories appeared globally coherent, except for the putative carnivore *Octopus* cf. *cynthiae* with a TP of only 2.5, a result that was improved with a variable trophic enrichment factor implying a TP of ~3.0 (Table 2). Similarly, the TP_Pima‐va_ value for *Ascidia* sp. (2.14) is coherent with its filter‐feeder strategy, whereas other TP estimates for that species could appear under‐ or overestimated. Overall, we did not find any evidence that a given feeding guild (herbivores, plankton‐feeders, detritivores, carnivores, etc.) was more sensitive than another to our comparison of TP estimates, i.e. the magnitude of differences between minimum and maximum TP values for those feeding guilds appeared independent of the baseline, trophic enrichment factor and/or calculation method we used.

**FIGURE 2:**
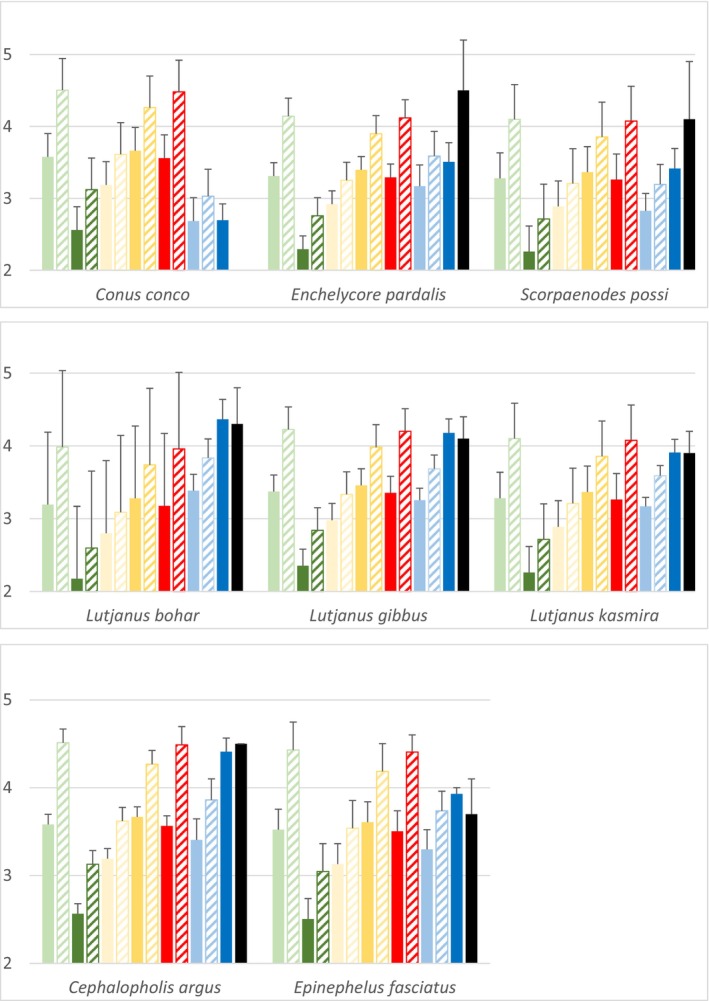
Trophic positions of eight mesopredators from the coral reefs of Marquesas Islands. Results obtained with primary producers are in green (light green: TP_algae_, light green hatched: TP_algae‐va_; dark green: TP_phyto_, dark green hatched: TP_phyto‐va_), those obtained with primary consumers are in gold (light gold: TP_Masp_; light gold hatched: TP_Masp‐va_; dark gold: TP_Pima_, dark gold hatched: TP_Pima‐va_), those obtained with source AA are in red (red: TP_AA‐sr_, red hatched: TP_AA‐sr‐va_), those obtained with combination of source and trophic AA are in blue (light blue: TP_AA glu‐phe (1)_, light blue hatched: TP_AA glu‐phe (2)_, dark blue: TP_AA tr‐sr_) and those coming from FishBase are in black (TP_ref_) (see text for TPs’ formulae). Vertical bars are standard deviations.

For the eight selected mesopredators, taking into account trophic amino acids confirmed the high variation in TPs, plus unrealistic results for TP_phyto_ and TP_phyto‐va_ with values around 2.2–2.7 for most of these species (Figure 2). TP_Glu‐Phe_(1), TP_Glu‐Phe_(2) and TP_Tr‐Sr_ values however added new information. TP_Glu‐Phe_(1) values were close to those obtained with TP_AA‐Sr_ (except for *C. conco*) and were always lower than those of TP_Tr‐Sr_; TP_Glu‐Phe_(2) being intermediate, except for *C. conco* (Figure 2). For five mesopredator fish, TP_Tr‐Sr_ values were relatively close to the TP_ref_ values from FishBase, but in two cases differences between these results were marked, i.e. ~1.0 for *Enchelycore pardalis* and ~0.7 for *Scorpaenodes possi* (Figure 2). However, TPs estimated with variable trophic enrichment factors (TP_algae‐va_, TP_AA‐Sr‐va_) produced results relatively close to TP_ref_ for these two species. In all cases for the eight studied mesopredators, the variable trophic enrichment factor (i.e. 2.5‰) resulted in higher TP estimates (increase of around 0.5–0.8) compared to those obtained with the conventional value (3.4‰) (Figure 2).

### 3.2 Differences between seasons

Since we considered that phytoplankton produced unrealistic TP values, we decided to explore the seasonal variation using only macroalgae and AA‐Sr as baselines, both with the conventional and variable values of trophic enrichment factor. The TPs calculated with macroalgae as the baseline were always higher in winter than in summer; among the 20 species analysed in both seasons, only one (*Ascidia* sp.) showed a non‐significant seasonal difference (Table 3). Differences in TPs between summer and winter were statistically significant and ranged from ~0.3 (*Spheciospongia* sp.) to ~1.2 TPs (*Scarus koputea*) when calculated with the conventional value of 3.4‰. However, the magnitude in seasonal differences changed with the variable trophic enrichment factors and showed less differences for herbivores (for instance, ~1.0 TP for *Scarus koputea*) and larger ones for carnivores, such as ~1.2 TPs for *Scorpaenodes possi* (Table 3).

**TABLE 3:** Mean trophic position (± SD) calculated with the Post' formulae with (A) macroalgae or (B) AA‐sources as baselines with the conventional enrichment factor, and with variable trophic enrichment factors (see text) for species having at least three individuals per season.

(A)		TP_algae_			TP_algae‐va_		
Fish		Summer	Winter	*p*‐Value	Summer	Winter	*p*‐Value
Acanthuridae	*Acanthurus nigricans*	2.24 ± 0.27	3.01 ± 0.24	<.001	1.98 ± 0.22	2.59 ± 0.19	<.001
	*Ctenochaetus marginatus*	2.62 ± 0.43	3.61 ± 0.30	<.001	2.28 ± 0.34	3.06 ± 0.24	<.001
Apogonidae	*Ostorhinchus relativus*	2.72 ± 0.13	3.71 ± 0.32	<.001	2.95 ± 0.15	4.01 ± 0.37	<.001
Cirrhitidae	*Cirrhitichthys oxycephalus*	2.95 ± 0.18	3.86 ± 0.27	<.001	3.65 ± 0.24	4.69 ± 0.37	<.001
Holocentridae	*Myripristis berndti*	2.72 ± 0.09	3.46 ± 0.25	<.001	2.95 ± 0.10	3.78 ± 0.28	<.001
Lutjanidae	*Lutjanus gibbus*	3.10 ± 0.07	3.97 ± 0.11	<.001	3.85 ± 0.05	5.04 ± 0.15	<.001
	*Lutjanus kasmira*	3.04 ± 0.18	3.94 ± 0.18	<.001	3.76 ± 0.25	5.00 ± 0.25	<.001
Muraenidae	*Enchelycore pardalis*	3.08 ± 0.06	3.87 ± 0.07	.008	3.82 ± 0.08	4.90 ± 0.10	.008
Pomacanthidae	*Centropyge flavissima*	2.94 ± 0.15	3.66 ± 0.21	<.001	No change		
Pomacentridae	*Chromis abrupta*	2.85 ± 0.12	3.78 ± 0.18	<.001	3.09 ± 0.14	4.16 ± 0.21	<.001
	*Lepidozygus tapeinosoma*	2.66 ± 0.14	3.60 ± 0.09	<.001	2.88 ± 0.16	4.06 ± 0.10	<.001
Scaridae	*Scarus koputea*	2.34 ± 0.35	3.59 ± 0.09	<.001	2.06 ± 0.28	3.05 ± 0.07	<.001
Scorpaenidae	*Scorpaenodes possi*	3.01 ± 0.25	3.87 ± 0.38	.039	3.73 ± 0.34	4.90 ± 0.52	.039
Serranidae	*Cephalopholis argus*	3.42 ± 0.11	4.07 ± 0.04	<.001	4.29 ± 0.15	5.17 ± 0.05	.005
	*Epinephelus fasciatus*	3.02 ± 0.28	3.98 ± 0.14	<.001	3.75 ± 0.38	5.05 ± 0.18	<.001
*Invertebrates*							
Ascidiidae	*Ascidia* sp.	1.94 ± 0.32	2.05 ± 0.43	.691	2.45 ± 0.50	2.68 ± 0.57	.556
Coniidae	*Conus conco*	3.23 ± 0.13	3.73 ± 0.21	.003	4.03 ± 0.17	5.16 ± 0.19	.001
Diadematidae	*Echinothrix diadema*	2.17 ± 0.21	3.04 ± 0.65	.024	1.93 ± 0.16	2.61 ± 0.53	.024
Diogenidae	*Ciliopagurus vakovako*	2.02 ± 0.18	2.46 ± 0.27	<.001	No change		
Spongidae	*Spheciospongia* sp.	2.24 ± 0.15	2.52 ± 0.35	.008	2.91 ± 0.23	3.34 ± 0.54	.002

(B)		TP_AA‐sr_			TP_AA‐sr‐va_		
Fish		Summer	Winter	*p*‐Value	Summer	Winter	*p*‐Value
Acanthuridae	*Acanthurus nigricans*	2.75 ± 0.27	2.24 ± 0.24	<.001	2.30 ± 0.22	1.98 ± 0.19	<.001
	*Ctenochaetus marginatus*	3.03 ± 0.43	2.74 ± 0.30	.177	2.61 ± 0.34	2.46 ± 0.27	.177
Apogonidae	*Ostorhinchus relativus*	3.13 ± 0.13	2.94 ± 0.32	.034	3.41 ± 0.15	3.20 ± 0.36	.034
Cirrhitidae	*Cirrhitichthys oxycephalus*	3.36 ± 0.18	3.10 ± 0.27	.004	4.21 ± 0.24	3.85 ± 0.37	.004
Holocentridae	*Myripristis berndti*	3.13 ± 0.09	2.69 ± 0.25	<.001	3.41 ± 0.10	2.92 ± 0.28	<.001
Lutjanidae	*Lutjanus gibbus*	3.50 ± 0.07	3.20 ± 0.11	.098	4.41 ± 0.09	4.00 ± 0.15	.076
	*Lutjanus kasmira*	3.44 ± 0.18	3.17 ± 0.18	.049	4.30 ± 0.25	3.95 ± 0.25	.053
Muraenidae	*Enchelycore pardalis*	3.48 ± 0.06	3.10 ± 0.07	.032	4.38 ± 0.08	3.86 ± 0.10	.032
Pomacanthidae	*Centropyge flavissima*	3.34 ± 0.15	2.90 ± 0.21	<.001	No change		
Pomacentridae	*Chromis abrupta*	3.26 ± 0.12	3.02 ± 0.18	<.001	3.56 ± 0.14	3.29 ± 0.21	<.001
	*Lepidozygus tapeinosoma*	3.07 ± 0.14	2.94 ± 0.09	.102	3.34 ± 0.16	3.20 ± 0.10	.102
Scaridae	*Scarus koputea*	2.75 ± 0.35	2.83 ± 0.09	.538	2.38 ± 0.28	2.44 ± 0.07	.538
Scorpaenidae	*Scorpaenodes possi*	3.42 ± 0.25	3.10 ± 0.38	.312	4.29 ± 0.34	3.86 ± 0.52	.312
Serranidae	*Cephalopholis argus*	3.73 ± 0.11	3.30 ± 0.04	.008	4.85 ± 0.15	4.13 ± 0.05	.005
	*Epinephelus fasciatus*	3.43 ± 0.28	3.21 ± 0.14	.018	4.30 ± 0.38	4.01 ± 0.18	.018
*Invertebrates*							
Ascidiidae	*Ascidia* sp.	2.34 ± 0.32	1.69 ± 0.43	.097	3.08 ± 0.50	1.97 ± 0.24	.008
Coniidae	*Conus conco*	3.64 ± 0.13	3.37 ± 0.21	.302	4.59 ± 0.17	4.37 ± 0.26	.302
Diadematidae	*Echinothrix diadema*	2.58 ± 0.21	2.28 ± 0.65	.325	2.25 ± 0.13	2.01 ± 0.53	.325
Diogenidae	*Ciliopagurus vakovako*	2.43 ± 0.18	1.80 ± 0.27	<.001	No change		
Spongidae	*Spheciospongia* sp.	2.64 ± 0.15	1.82 ± 0.17	<.001	3.54 ± 0.23	2.27 ± 0.26	<.001

*Note*: Significance (*p*‐value) of seasonal differences assessed with non‐parametric Kruskal–Wallis test. No change = usual 3.4‰ enrichment conserved for omnivores.

The TPs calculated with AA‐Sr displayed a different pattern, with eight cases without significant seasonal differences, irrespective of the conventional or variable enrichment factors (Table 3). More importantly, the TPs were always higher in summer than in winter, which is the opposite of what we obtained using macroalgae as baseline. Significant differences in TPs between summer and winter ranged from ~0.2–0.3 (*Epinephelus fasciatus*) to ~0.8–1.3 TPs (*Spheciospongia* sp.), depending on the value of the trophic enrichment factor (Table 3). Globally, the highest seasonal variability concerned low‐trophic rank species such as the primary consumers *Ascidia* sp. and *Spheciospongia* sp., whereas lower variabilities were observed for high‐trophic rank species (e.g. *C. conco*, *E. fasciatus*).

## 4 DISCUSSION

In this study, we found that the estimation of TPs is extremely sensitive to the formula employed, to the baseline, to the value of the trophic enrichment factor used and to the method (i.e. BSIA versus CSIA). Overall, our results raise technical and ecological issues and call for the development of novel approaches that go beyond the use of ‘ready‐to‐use’ formulas for TP calculation to better assess the ecological realities of trophic positions of species within ecosystems.

### 4.1 Is it really possible to make a ‘good choice’ among available methods?

While this question may seem trivial, it remains a key point in trophic ecology. Given the great functional variability within ecosystems, and the complex interactions between the species that compose them, it is reasonable to suggest that there is no single and clear answer to this question. An examination of our various results clearly points in this direction, and it would be highly speculative, if not false, to conclude that a particular method of calculating TP with a well‐defined trophic enrichment factor is consistently the best way to proceed, whatever the season or trophic category of the species concerned.

The interpretation of the TPs estimated from Post's equation (2002a) and the bulk δ^15^N values of the organisms is often complex, because the estimation depends on the variation in the isotopic nitrogen composition of primary producers and the number of trophic levels between consumers and the baseline (Vander Zanden & Rasmussen, 2001). To reduce the risk of idiosyncratic temporal and spatial variability of the δ^15^N values for the baseline, primary consumers can preferentially be used to estimate the isotopic composition of the baseline. These organisms (both grazer and filter‐feeding species in our study) can however present a certain degree of omnivory, thereby assuming a TP higher than 2 (Vander Zanden & Fetzer, 2007). Such discrepancies might explain why few TP values we obtained with primary consumers showed TPs <2 in some consumers.

Another potential source of error is related to the ^15^N enrichment factor of 3.4‰ (Post, 2002a), which is known to be biased, especially for higher trophic levels (Hussey et al., 2014). Indeed, some authors propose to use different enrichment factors depending on the trophic groups considered, particularly for herbivores (Caut et al., 2009; Hussey et al., 2014; Martínez Del Rio et al., 2009; Vanderklift & Ponsard, 2003). Our results support this suggestion because we obtained TP estimates with variable trophic enrichment factors that appeared more relevant to the ecological theory. For instance, we found TPs closer to ~2.1–2.5 for *Acanthurus* spp. with macroalgae as baseline, compared with TPs obtained with the 3.4‰ conventional value. Similarly, higher TPs were obtained for carnivores with an enrichment factor of 2.5‰ and sometimes with TPs higher than those referenced in FishBase. Although the ^15^N enrichment factor of 3.4‰ has been criticized, it is still largely used for practical reasons, such as a lack of empirical data that prevent the assessment of more realistic enrichment factors adapted to the species to be studied. Even so, it is relatively easy to test different enrichment factor values, higher for herbivores and lower for carnivores, even in the absence of precise data, in order to avoid as far as possible the 3.4‰ conventional value whose imprecision is becoming increasingly apparent. More controlled feeding experiments and modelling work are needed to fill this gap of knowledge and propose a widely applicable and accepted approach.

According to the fish diet data available in FishBase (Froese & Pauly, 2018), the TP_ref_ of our seven mesopredator fish should be between 3.7 and 4.5. The TP estimates based on the δ^15^N values of the source and trophic AAs (TP_Tr‐Sr_), as recommended by Choy et al. (2015), presented the results closest to the TP_ref_, but only for five species. Interestingly, TP estimates from FishBase are also relatively close to those obtained with macroalgae as baseline and with an enrichment factor adapted to carnivores (2.5‰ in our case) for four of our seven fish species, a pattern that was also found with source AAs and an adapted enrichment factor. Calculations based on the δ^15^N of glutamic acid and phenylalanine, taking into account the *β* and Δ values proposed by Chikaraishi et al. (2010), i.e. TP_gly‐phe_ (1) and (2) values, underestimated the TPs of consumers even more. For the constant *β*, representing the difference between the δ^15^N of source and trophic amino acids in primary producers, the value of 3.4‰ is commonly accepted (Chikaraishi et al., 2009, 2010; Hannides et al., 2013; McCormack et al., 2019; Vokhshoori & McCarthy, 2014). Concerning the constant Δ, which represents the trophic enrichment in ^15^N between the source and trophic amino acids of consumers, previous work on a limited number of organisms, type of tissues and physiological conditions proposed the value of 7.6‰ (Chikaraishi et al., 2009). However, several studies concluded that this value produces underestimated trophic positions (Dale et al., 2011; Germain et al., 2013; Lorrain et al., 2009, 2015). Accordingly, controlled feeding experiments are needed to establish appropriate enrichment factors and to evaluate the amino acid turnover rates (Bradley et al., 2014). Studies should also be carried out to better understand the mechanisms associated with the isotopic fractionation factor, itself linked to amino acid metabolism (e.g. enzymatic transamination of glutamic acid; Miura & Goto, 2012), and to compare the estimated TPs with techniques other than amino acid analysis. Assessing TPs through the nitrogen composition of source and/or trophic amino acids is often considered as a powerful method (Chikaraishi et al., 2009; Nielsen et al., 2015; Sackett et al., 2015) but it suffers from relatively high costs (up to ~100–110€ per sample versus usually ~8–10€ per sample for bulk δ^15^N) that likely limit its wider use.

### 4.2 The role of the baseline

The importance of the baseline is already apparent in the previous section but it should be deeply discussed. By comparing with the estimates carried out according to the method of Post (2002a), the calculations taking the δ^15^N of the macroalgae (TP_algae_) and of *Pinctada margaritifera* (TP_Pima_) at the baseline give several results that were relatively close to the TP_ref_ or putative feeding guilds and the estimates obtained with amino acids in general. However, our results also highlighted a marked underestimation of the TPs when the δ^15^N values of phytoplankton (TP_phyto_ and TP_phyto‐va_) are used. This remains unclear because there is no doubt that phytoplankton is an important source of organic matter in Marquesas Islands, through pelagic–benthic coupling processes (Fey et al., 2020, 2021) and several of our invertebrates and fish species likely at least partly rely on pelagic organic matter, such as planktivores or filter‐feeders. However, one cannot exclude the possibility that, despite sampling and analytical precautions, this phytoplankton compartment actually also contains a part of non‐autotrophic biological material (heterotrophic bacteria, micro‐zooplankton, etc.). This would partly explain the high δ^15^N values obtained for the phytoplankton, and consequently the unrealistic TP_phyto_ (and TP_phyto‐va_) values found for many consumers.

Comparing the estimates of fish TPs obtained through various methods with the TP_ref_ based on stomach content analyses (Froese & Pauly, 2018) revealed that part of our results are consistent with those referenced in FishBase, in particular when using TP_AA‐Sr_. This is the case, for instance, not only for corallivore or some zooplanktivore species, but also when using TP_algae‐va_ or TP_AA‐Sr‐va_ for some herbivores or some carnivores (Table 2). However, in most cases our results suggest that the values proposed by FishBase (Froese & Pauly, 2018) over‐ or underestimated TP of fish. Our observations are consistent with those of Page et al. (2013) who suggest that TP estimates based on stomach content analyses tend to badly reflect the ecological reality of TPs. For example, invertebrates, herbivore and omnivore fish would likely contribute more significantly to the diet of carnivore and piscivore species, compared to what is suggested by stomach content analyses alone. For instance, stomach contents do not have to reflect assimilation of prey, can overestimate prey with hard parts and underestimate easily digestible prey like jellyfish, polychaetes or some palatable algae (Carassou et al., 2008; Letourneur et al., 2013). In addition, gut contents are a snapshot of diet that is much more temporally limited, and thus likely variable, than tissue isotopes that integrate over time (Vander Zanden et al., 1999).

Fey (2019) and Fey et al. (2021) compared the food webs between the Marquesas Islands and Mururoa, a French Polynesian atoll (Page et al., 2013); both food webs being studied with macroalgae as baseline and using the Post (2002a) formulae. Overall and except for a few cases such as *Ctenochaetus* spp. and *Scarus* spp., the TPs did not show marked differences between these two food webs. However, the organisms that make up Marquesan food webs show higher than usual δ^15^N signatures on coral reefs (Fey et al., 2021). For example, in the Marquesas Islands, macroalgae have a mean δ^15^N value of 11.6 ± 0.9‰ compared to 2.8 ± 0.3‰ in Mururoa (Page et al., 2013), or even from 0.4 ± 1.7‰ to 5.2 ± 1.6‰ in New Caledonia (Briand et al., 2015). Despite the potential biases of the choice of a given baseline, these results highlight the importance of TP estimates in order to be able to take into account the variability of the baseline's δ^15^N values (Cabana & Rasmussen, 1996; Post, 2002b; Vander Zanden & Rasmussen, 1999). However, one weakness of TP estimates is the use of a single baseline value. Indeed, most consumers acquire nitrogen from several food webs, feeding on both benthic/littoral (e.g. macroalgae, seagrass, terrestrial detritus) and pelagic (e.g. phytoplankton) sources (Briand et al., 2016; Fey et al., 2021; Quezada‐Romegialli et al., 2018). In our study, this bias is likely circumvented by the use of the δ^15^N of the source amino acids (δ^15^N_AA‐Sr_) analysed on mesopredators of high trophic ranks. Knowing that predators consume a wide variety of prey probably based on different sources of organic matter, their δ^15^N_AA‐Sr_ is assumed to reflect the ‘global’, averaged baseline.

### 4.3 Ecological implications of seasonality

The use of the δ^15^N_AA‐Sr_ values and δ^15^N_algae_ values specific to both seasons highlighted clear seasonal differences in TP of consumers. Moreover, we found an opposite seasonal trend according to the baseline employed. This latter aspect can be explained by differences in δ^15^N values between seasons (δ^15^N_algae_ were lower in winter, whereas δ^15^N_AA‐Sr_ were higher in winter), stressing the importance of the choice of baseline. If we assume that δ^15^N_AA‐Sr_ better reflect the global baseline than δ^15^N_algae_, this implies an overall ^15^N enrichment of the food web in summer (Fey et al., 2021). This opposite seasonal trend between TPs obtained with δ^15^N_AA‐Sr_ and δ^15^N_algae_ values may be due to a temporal lag in turnover processes. The δ^15^N_AA‐Sr_ values were measured on consumers, which likely have a longer turnover than primary producers, and a renewal time of the muscle tissues analysed roughly estimated to ~3 months before sampling (Vander Zanden et al., 2015). Conversely, the δ^15^N values of the baseline obtained with bulk data reflected recent variations of isotope composition of the organic matter (OM) sources at the time of collection, i.e. summer or winter.

Temporal variations in TPs have already been detected in other marine areas, for example for zooplankton in the California Current Ecosystem during El Niño period (Décima et al., 2013). Hannides et al. (2009) also showed changes in δ^15^N of 10‰ for zooplankton in the North Pacific subtropical gyre, depending on the sampling period. These variations would not only reflect changes in δ^15^N of nutrients available in the environment (Fey et al., 2021), but could also be linked to changes in the trophic position of species within zooplankton in connection with changes in phytoplankton communities on which they feed (Hannides et al., 2009). The discrepancy between the summer and winter TPs of species also suggests a certain degree of feeding plasticity among consumers. This could be related to a lower abundance/density of macroalgae and other benthic sources of organic matter (for benthic feeders) or phytoplankton (for filter‐feeders) in summer (Galzin et al., 2016). This thus could generate a partial shift in food research effort towards primary–secondary consumers (invertebrates, fish; for benthic feeders) or towards bacteria (for filter‐feeders), which would be the cause of the summer increase in TPs.

In conclusion, we have shown in this study that the evaluation of TP remains a real challenge in ecology because no calculation formula emerges clearly as being systematically the most suitable. In addition, taking into account the baseline and its temporal variations as well as variable trophic enrichment factors, adapted to various feeding guilds, makes the scope of the issue even more complex. Despite analytical progress and technical developments, each situation must be assessed on a case‐by‐case basis, requiring expert knowledge of ecosystems, their local environmental conditions and the species that inhabit them to avoid inappropriate calculations and hazardous interpretations.

## AUTHOR CONTRIBUTIONS

**Yves Letourneur:** Conceptualization (lead); data curation (equal); formal analysis (equal); funding acquisition (lead); investigation (equal); methodology (equal); project administration (lead); resources (lead); software (equal); supervision (lead); validation (lead); visualization (equal); writing – original draft (equal); writing – review and editing (lead). **Pauline Fey:** Conceptualization (equal); data curation (lead); formal analysis (equal); investigation (equal); methodology (equal); software (lead); writing – original draft (equal); writing – review and editing (equal). **Jan Dierking:** Funding acquisition (supporting); investigation (supporting); methodology (supporting); writing – original draft (supporting); writing – review and editing (equal). **René Galzin:** Conceptualization (equal); funding acquisition (supporting); investigation (equal); project administration (supporting); supervision (equal); writing – original draft (supporting); writing – review and editing (supporting). **Valeriano Parravicini:** Conceptualization (supporting); data curation (supporting); formal analysis (supporting); funding acquisition (supporting); investigation (equal); methodology (equal); project administration (supporting); resources (supporting); supervision (supporting); validation (supporting); writing – original draft (equal); writing – review and editing (supporting).

## CONFLICT OF INTEREST STATEMENT

The authors declare no competing interests.

## Data Availability

All data necessary to reproduce the results and figure are publicly available on https://doi.org/10.5061/dryad.2fqz612vw.
